# Multiple Comparisons of the Efficacy and Safety for Seven Treatments in Tibia Shaft Fracture Patients

**DOI:** 10.3389/fphar.2019.00197

**Published:** 2019-04-09

**Authors:** Haibo Li, Dapeng Yu, Shaobin Wu, Yihang Zhang, Liang Ma

**Affiliations:** ^1^Department of Orthopaedic, Tianjin Hospital, Tianjin, China; ^2^Department of Emergency Trauma Surgery, the Wendeng Osteopathic Hospital, Weihai, China; ^3^Department of Orthopaedic, Weifang Traditional Chinese Hospital, Weifang, China; ^4^Graduate Student Education Center, Shandong Academy of Medical Sciences, Jinan, China; ^5^Department of Orthopaedic, Affiliated Hospital of Shandong University of Chinese Medicine, Jinan, China

**Keywords:** tibia shaft fracture, randomized controlled trial (RCT), network meta-analysis (NMA), efficacy, safety

## Abstract

**Background:** A tibia shaft fracture is one of the most common long bone fractures, with two general types, open fracture and close fracture. However, there is no universally accepted guideline suggesting which treatment to use under certain circumstances. Therefore, a comprehensive network meta-analysis (NMA) is needed to summarize existing studies and to provide more credible data-based medical guidelines.

**Methods:** Available literature was identified by searching medical databases with relevant key terms. Studies that met the inclusion and exclusion criteria, baseline, intervention, and the outcome of treatments, were extracted. A comparative connection of these studies was demonstrated through net plots. Continuous variables and binary variables were reported as mean difference (MD) and odds ratio (OR) with a 95% credible interval (CrI), respectively. The comparison of direct and indirect outcome and their *P*-value were listed in the node-splitting table. Treatments for each endpoint were ranked by their surface under the cumulative ranking curve (SUCRA) value. A heat plot was created to illustrate the contribution of raw data and the inconsistency between direct and indirect comparisons.

**Results:** According to the search strategy, 697 publications were identified, and 25 records were included, involving 3,032 patients with tibia shaft fractures. Seven common surgical or non-surgical treatments, including reamed intramedullary nailing (RIN), un-reamed intramedullary nailing (UIN), minimally reamed intramedullary nailing (MIN), ender nailing (EN), external fixation (EF), plate, and cast, were compared, in terms of time to union, reoperation, non-union, malunion, infection and implant failure. Plate performed relatively better for time to union, while cast might be the best choice in close cases to reduce the risks of reoperation, non-union, malunion, and infection. To prevent implant failure, EN seemed to be better.

**Conclusion:** Cast might have the highest probability of the most optimal choice for tibia shaft fracture in close cases, while reamed intramedullary nailing ranked second.

## Introduction

The tibia is a large bone in the lower extremity other than the fibula. A tibia shaft fracture is the most common type of long bone fractures. According to vast clinical data, the causation of diaphyseal fractures of the tibia is usually grouped into two types, bending load and torsion (Johner and Wruhs, 1983). Bending load, led by direct impact injuries, generally of high-velocity trauma, like a car accident, is an increasing fracture cause in modern life and most cases include open fractures with broken skin, even exposed bone. While torsion, low-energy injuries, like falls or sports injuries, is a common cause of closed fractures (Grutter et al., 2000). Pain and regional swelling are clear signs and symptoms. Deformity, distortion and angulation can also be observed (Delee and Stiehl, 1981). In light of AT/OTA, a Comprehensive Classification of Fractures of the Long Bones proposed by the American Orthopedic Trauma Association (OTA), there are three classes: A indicates simple cases, including spiral, oblique (≥30°) and transverse (<30°) fractures; B are fractures with a third or more fragments, but still with contact, like intact wedge fracture, fragmentary wedge fracture; C embodies intact and fragmentary segmental fractures, belonging to multifragmentary fractures (Swiontkowski et al., 2000). With the help of X-rays, computed tomography (CT) scans, magnetic resonance imaging (MRI) scans, and other tests, the type of fracture can easily be confirmed. Nearly two in every thousand people have suffered a tibia fracture (Alho et al., 1992). Although osteoporosis is a contributing factor, accidents are the major culprits, since the average age of patients are around 37 years, while teenage males are the most vulnerable population (Court-Brown and Mcbirnie, 1995).

In general, a non-surgical treatment such as a cast, is applied for closed and simple fractures, while surgical treatments are suggested for patients with open or severe fractures (Littenberg et al., 1998; Busse et al., 2008). However, in clinical practice, these treatments can be uncertain. Choices are more difficult due to differences in efficacy, safety, invasion, and adaptation to different treatments, as well as complex patient situations. Even in the scope of surgical management, the existence of so many technologies, such as reamed, un-reamed, minimally reamed intramedullary nailing (MIN) and etc., provides physicians with more choices but also causes more confusion at the same time (Henley et al., 1998). Although a mass of studies on the comparison of different treatments for tibia shaft fractures have been published, quality issues, and inherent problems of experimental design, restrict the application of their outcomes. As indicated by the Study to Prospectively Evaluate Reamed Intramedullary Nails in Patients with Tibia Fractures (SPRINT), a blinded randomized trial, a potential benefit of un-reamed intramedullary nailing (UIN) over reamed intramedullary nailing (RIN) for open fracture patients was revealed, although this difference was not significant (Bhandari et al., 2008). However, another report regarded RIN as a better treatment to manage open fractures, considering that only 9% of patients treated with RIN had implant failure, while 29% had implant failure for those treated with UIN; while for other endpoints, no significant difference was observed (Keating et al., 1997). Therefore, a comprehensive network meta-analysis (NMA) is required due to these contradictions, to provide more informative suggestions.

This analysis compared the efficacy and safety of seven treatments, including RIN, UIN, MIN, Ender nailing (EN), external fixation (EF), plate (P), and cast (C), in terms of their performance on time to union, the incidence of reoperation, non-union, malunion, infection, and implant failure, in order to provide supporting evidence for a reasonable choice for the treatment of tibia shaft fractures.

## Materials and Methods

### Search Strategy

To identify available literature from electronic databases, including but not limited to Cochrane Library, Embase and PubMed, the following Medical Subject Headings and their synonyms were used jointly, as disease “tibia shaft fracture”; type of literature “randomized controlled trial,” “quasi-randomized trial,” or “meta-analysis; treatment “intramedullary nailing,” “Ender nailing,” “external fixation,” “plate,” “cast,” etc.; any endpoint “time to union,” “reoperation,” “nonunion,” “malunion”; “infection,” “implant failure,” or “compartment syndrome,” References listed in relevant meta-analyses or systematic reviews were checked manually in order to spot potential trials.

### Inclusion and Exclusion Criteria

The inclusion criteria: (i) The design of included trials must be a randomized controlled trial (RCT) or quasi-randomized trial (QRT); (ii) Patients in selected studies must be diagnosed with a tibia shaft fracture, with no extra requirement on its severity or type; (iii) The intervention of two arms must be a placebo (PBO) or any surgical or non-surgical treatments of a tibia shaft fracture; (iv) At least one endpoint for each group should be reported with specific value. Additionally, studies that met the inclusion criterion were excluded if: (i) follow-up was <6 moths; (ii) phase II or III clinical trials on unlisted intervention and trials whose studied treatment cannot form a loop with others; (iii) trials fixed on the comparison of particular surgical methods of an identical treatment. Among the ten studies excluded due to insufficient connections, four articles were not consistent with the design type, three articles were not consistent with the outcome index, and the other three articles did not satisfy the publication type requirement and did not provide endpoint data (Table S1).

### Data Extraction and Outcome Measure

Two investigators independently reviewed the full manuscripts of eligible studies and extracted information, including basic information of study, baseline patients' characteristics, intervention of each arm, and endpoints. Author, country, year of publication, the design of trial, and the length of follow-up was recorded. Age, gender ratio, and type of fracture was extracted as the patients baseline information, for each group.

Time to union is a useful outcome to assess the treatment of a tibia shaft fracture. For low energy fracture, the time to union ranges from 10 to 13 weeks, and for high energy fracture, the time is much longer and ranges between 13 and 20 weeks. A shorter time to union usually indicates a higher efficacy. Secondary surgery is often required for tibia shaft fractures and reoperation rates are also an objective endpoint to measure the treatment (Harris and Lyons, 2005). Non-union, malunion and infection, involving deep infection and superficial infection, are common complications and is regarded as an important safety index. Non-union is related to a permanent failure of fracture healing and malunion indicates an improper healing in which bone is twisted or bent (Alho et al., 1993; Milner et al., 2002). Infection is a possible problem after any surgical procedure, especially in open fracture cases with large scaled exposed injuries (Kulshrestha, 2008). The loosening or breakage of the internal fixation device is the general cause of implant failure, which interferes with the curative effect of the treatment (Esan et al., 2014). As uncommon endpoints, that were only found in few articles, were ignored, the remaining six end points included were all comparable outcomes. For example, endpoints related with time to union or healing, were excluded because of inconsistent criteria and insufficient data. Additionally, endpoints related to pain problems such as VAS pain score and Knee pain numbers were excluded, due to insufficient data in <5 publications.

### Statistical Analysis

Among six outcomes, except for time to union, which is a continuous variable, the remaining five are all discrete variables. The mean difference (MD) and odds ratio (OR) were therefore introduced to evaluate the differences among interventions on continuous variables and dichotomous variables, respectively, and 95% credible interval (CrI) was calculated as well to examine the significance. For MD, 95% CrI including 0, and for OR, including 1 were considered as insignificant difference.

The traditional meta-analysis was first conducted with only direct evidence fitting a fixed-effects model, to estimate the heterogeneity by *Cochran's Q* method and *I squared statistic*. If *P*_*h*_ < 0.05 or *I*^2^ > 50%, a significant heterogeneity was observed, and the random model was altered. After that, both direct and indirect data were combined and a NMA was gained using the statistical software R (Version 3.4.1).

The connection between each intervention for every endpoint was demonstrated by the net plot. The size of the node in the net plot reflected the number of involved patients and the line width indicates the quantity of each trial. The slash table displayed the results of NMA and the node-splitting table exhibited the comparison between direct and indirect data, in which the associated *P*-value <0.05 indicated a significant inconsistency between direct and indirect evidence. Additionally, the surface under the cumulative ranking curve (SUCRA) was estimated to rank the treatment for each outcome. Moreover, a heat plot was generated to show the contribution of direct evidence to the combined results and the inconsistency between direct and indirect evidence. Eventually, publication bias was analyzed and plotted in the comparison adjusted funnel. An additional subgroup analysis was conducted, stratified by the type of fracture, as open and closed cases.

## Results

### Literature Identification

**Figure 1:**
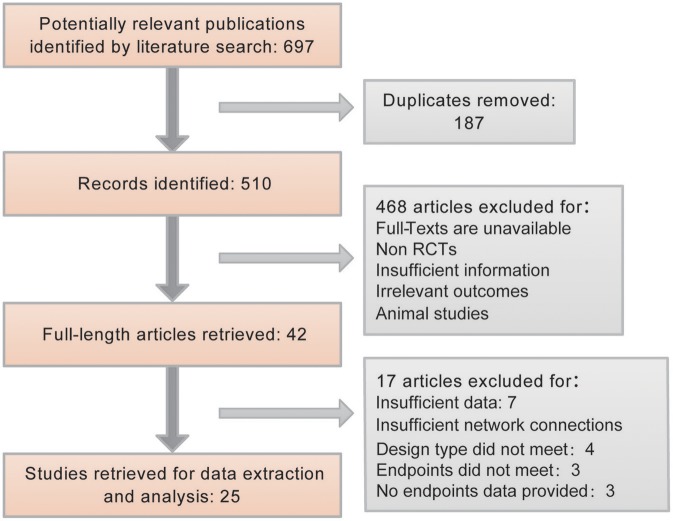
Flow chart for the process of screening out the included studies.

In accordance with the search strategy, 697 publications were identified from electronic medical databases. After removing 187 duplicates, the remaining 510 literature results were screened. Among them, only 42 met the inclusion standards. The remaining 468 other studies were animal-based studies, non-RCTs, and studies with insufficient information or irrelevant outcomes. Furthermore, another 17 articles were excluded because of insufficient data or network connections. We included RCTs and QRTs because we required sufficient data to accomplish a good NMA study. When only RCT articles were included, the number of references did not support the analysis. Finally, 25 studies were included and used to provide primary data for further analysis. The process is explained using a flow chart in Figure 1.

### Characteristics of Included Trials

**Figure 2:**
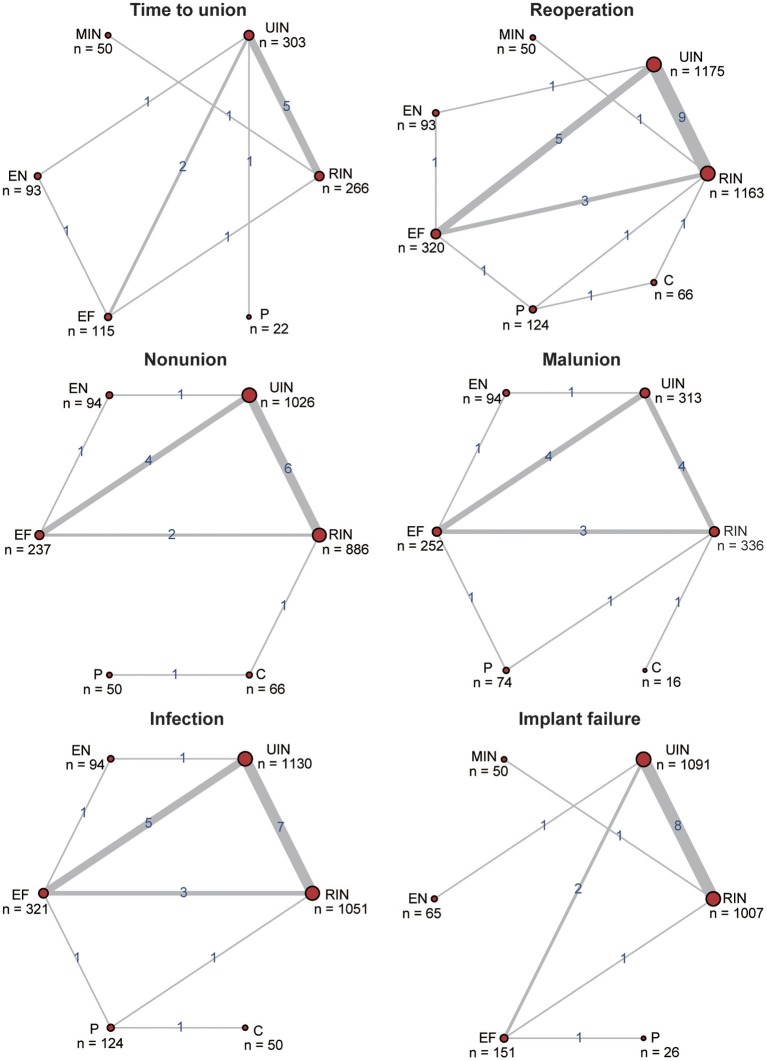
Net plots for six efficacy endpoints. The efficacy endpoints include time to union, reoperation, non-union, malunion, infection, and implant failure. The net plots show the direct comparison of different treatments, with node size corresponding to the sample size. The number of included studies for specific direct comparison determines the thickness of solid lines.

A total of 25 RCTs or QRTs published between September 1979 and October 2016 served as the data source, with 3,032 tibia shaft fracture patients, including 1,347 open cases and 1,685 closed cases. In agreement with the epidemiology, the average age of all subjects was 37 years, while males constituted the high-risk group (78.12%). The details of each included trial are listed in Table 1. For all six endpoints, the comparison between UIN and RIN was the most commonly reported, while EF and UIN was the second most commonly reported. The incidence of reoperation was recorded in all seven treatments. More information about direct evidence can be found in Figure 2. The evaluation results of overall quality of included studies are provided in Figure S1. QRT was assessed as a high risk, which could not be blinded by subjects and doctors due to the limitation of treatment methods, but could be blinded by researchers.

**Table 1:** Main characteristics of included studies.

**Author, year, location**	Design^**^		**Severity**	**Group 1**					**Group 2**					Outcomes^***^
		**Follow-up**		Treatment^*^	**Age/years**	**Male (%)**	**Size**	Size (O/C)^**^	**Treatment**	**Age/yrs**	**Male (%)**	**Size**	**Size (O/C)**	
Bach and Hansen, 1989, USA	QRT	16 m	Gustilo 2/3	P	37 (14–71)	–	26	26/0	EF	37 (14–71)	–	30	30/0	②④⑤⑥	
Bhandari et al., 2008, Multi	RCT	12 m	Gustilo 1–3B, AO A-C	RIN	39.1 ± 16.1	73.5	622	206/416	UIN	39.8 ± 15.9	74.0	604	194/410	②③⑤⑥	
Blachut et al., 1997, Canada	QRT	12 m	ISS 9	RIN	35	–	73	0/73	UIN	35	–	63	0/63	②③④⑤⑥	
Braten et al., 2005, Norway	QRT	12 m	Gustilo 1/2	EF	41 (16–83)	–	41	7/34	RIN	43 (16–90)	–	38	6/32	①②④⑤	
Choudary and Kanthimathi, 2012, India	RCT	12 m	AO A-C	RIN	25 (21–30)	80	20	6/14	UIN	25 (21–30)	77.8	18	3/15	①②③⑤	
Court-Brown et al., 1996, England	QRT	12 m	AO A-C A 23	RIN	35	78	25	0/25	UIN	36.1	68	25	0/25	①②③④⑥	
Fernandes et al., 2006, Brazil	QRT	12 m	A20	UIN	34	82.6	23	0/23	P	34	86.4	22	0/22	①	
Finkemeier et al., 2000, USA	RCT	19 m	AO B-C	RIN	33.8 (16–88)	78.7	44	19/25	UIN	33.8 (16–88)	78.7	50	26/24	②⑥	
Gaebler et al., 2011, Austria	RCT	12 m	Gustilo 1–3A	RIN	39 ± 13	64	50	0/50	MIN	36 ± 15	68	50	0/50	①②⑥	
Henley et al., 1998, USA	QRT	495 d	AO A-C	UIN	33 (14–81)	79	104	104/0	EF	33 (16–77)	75.7	70	70/0	②③⑤⑥	
Holbrook et al., 1989, USA	QRT	18.5 m	Gustilo 2–3B, AO A-C	EF	25 (7–65)	–	28	28/0	EN	28 (15–66)	–	29	29/0	①②③④⑤	
Inan et al., 2007, Turkey	QRT	43.3 m	Gustilo 1/2	UIN	31.7 (17–54)	82.8	29	29/0	EF	32.3 (15–64)	87.5	32	32/0	①②③④⑤	
Keating et al., 1997, Canada	RCT	22 m	Gustilo 3A	RIN	37 (16–88)	84.6	50	50/0	UIN	37 (16–88)	84.6	44	44/0	①②③④⑤⑥	
Larsen et al., 2004, Norway	RCT	3.8 y	Gustilo 1–3B	RIN	41 (15–87)	59.1	22	1/21	UIN	47.5 (18–81)	52.2	23	7/16	①②③④⑥	
Mohseni et al., 2011, Iran	RCT	12 m	Gustilo 1–3A	EF	28.92 ± 8.88	88	25	25/0	UIN	31.8 ± 5.24	80	25	25/0	②③④⑥	
Obremskey et al., 2017, USA	QRT	12 m	Gustilo 3A/3B	RIN	41.9 ± 15.6	71	17	0/17	C	43.2 ± 14.3	68	15	0/15	②③④	
Ramos et al., 2014, Sweden	RCT	12 m	AO 42 A1-3,B1	RIN	38 (19–70)	30	27	2/25	EF	46 (18–71)	29	31	9/22	②③④⑤⑥	
Rodrigues et al., 2014, Brazil	RCT	12 m	Gustilo 1/2, AO A-C	RIN	30.5 ± 2.0	92.3	26	26/0	EF	30.3 ± 2.2	90.3	31	31/0	②③④⑤	
Sadighi et al., 2011, Iran	RCT	6 m	Gustilo 1–3A	RIN	40.24 ± 12.32	80	30	0/30	UIN	38.42 ± 14.28	76	30	0/30	②⑤⑥	
Soleimanpour et al., 2008, Iran	RCT	9 m	AO C	UIN	38.4 ± 14.1	82.1	67	45/22	EN	37.5 ± 12.1	73.4	64	30/34	①②③④⑤	
Tabatabaei et al., 2012, Iran	RCT	–	Gustilo 1–3B	RIN	26.4 (20–40)	90	61	61/0	UIN	26.9 (20–45)	88	59	59/0	①②⑤⑥	
Tornetta et al., 1994, USA	QRT	21 m	Gustilo 1–3A	UIN	41 (21–73)	73.3	15	15/0	EF	37(19–86)	64.3	14	14/0	①②④⑤	
Tu et al., 1995, China	QRT	20.5 m	Gustilo 3B	UIN	38.5 (16–65)	83.3	18	18/0	EF	38.5 (16–65)	83.3	18	18/0	②③④⑤⑥	
Vallier et al., 2011, USA	RCT	19.9 m	Gustilo 3A/3B	P	38.5 ± 14.7	83.3	48	19/29	RIN	38.1 ± 12.6	80.4	56	21/35	②④⑤	
van der Linden and Larsson, 1979, Sweden	RCT	–	AO 42 A-C	P	–	70	50	6/44	C	–	70	50	6/44	②③⑤	

* *RIN, reamed intramedullary nailing; UIN, un-reamed intramedullary nailing; MIN, minimally reamed intramedullary nailing; EN, Ender nailing; EF, external fixation; P, plate; C, cast*.

** *RCT, randomized controlled trial; QRT, quasi-randomized trial; O, open; C, closed*.

*** *Outcomes: time to union; reoperation; nonunion; malunion; infection; implant failure*.

### Time to Union

**Figure 3:**
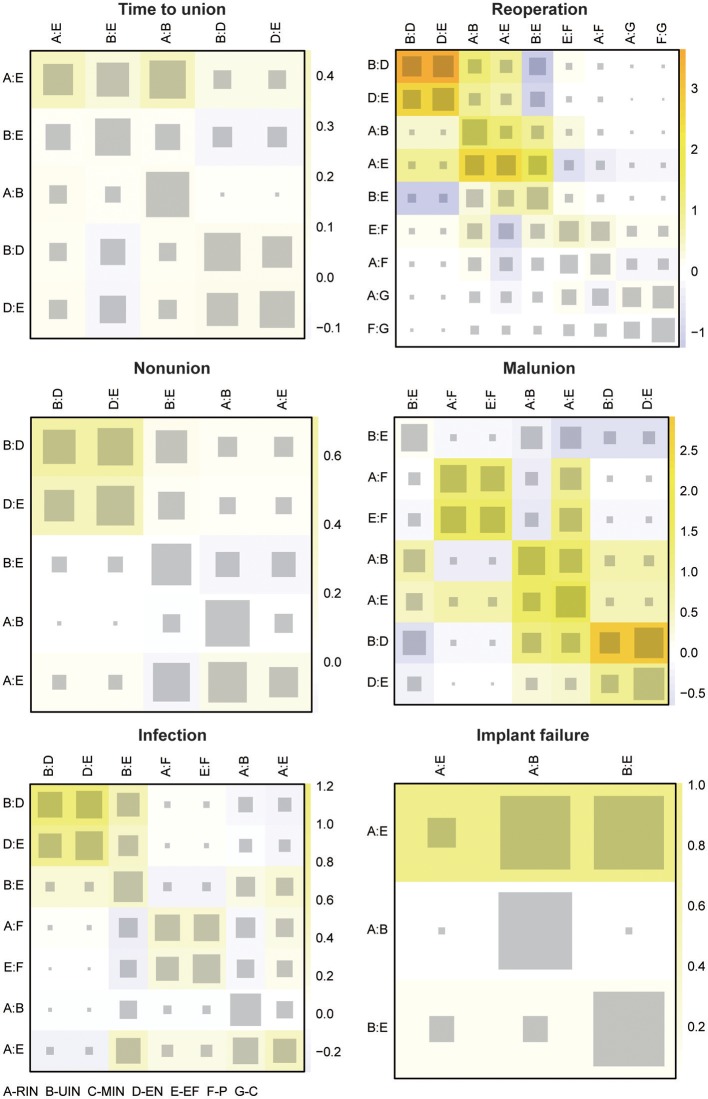
Heat plots for six efficacy endpoints. The efficacy endpoints include time to union, reoperation, non-union, malunion, infection, and implant failure. The size of the gray squares indicates the contribution of the direct evidence (shown in the column) to the network evidence (shown in the row). The colors are associated with the change in inconsistency between direct and indirect evidence (shown in the row). Blue colors indicate an increase of inconsistency and warm colors indicate a decrease.

Time to union is the only continuous variable among all six endpoints. It is a good indicator for treatment efficacy, although it is seriously influenced by the severity of a fracture. According to the direct, indirect and combined results listed in Tables 2, 3, no treatment significantly exceeded others for all associated 95% CrI containing 0. Although P had a positive MD value, which meant less union time was required, in contrast to other five interventions (RIN vs. P MD: 1.01, 95% CrI −8.67 to 10.62; EN vs. P MD: 1.89, 95% CrI −8.95 to 13.08; MIN vs. P MD: 3.00, 95% CrI −10.13 to 15.95; UIN vs. P MD: 4.23, 95% CrI −4.64 to 13.17; EF vs. P MD: 4.36, 95% CrI −5.52 to 14.67), none of them showed statistical significance. Similar to the combined outcome, SUCRA results shown in Table 4 indicate that P (0.609) was ranked at first place, RIN (0.601) and EN (0.489) second and the third, respectively. No significant inconsistency and publication bias was observed in Figure 3 and Figure S2.

**Table 2:** Network meta-analysis results for six efficacy endpoints.

Time to union^*^	**RIN**	2.12 (0.77, 5.93)	1.63 (0.05, 48.42)	4.01 (0.33, 54.05)	1.90 (0.55, 7.03)	1.97 (0.22, 17.46)	0.52 (0.03, 8.76)	**Reoperation**
	−3.21 (−7.01, 0.45)	**UIN**	0.77 (0.02, 26.84)	1.88 (0.17, 22.42)	0.89 (0.28, 3.03)	0.93 (0.09, 9.21)	0.24 (0.01, 4.81)	
	−1.99 (−10.56, 6.72)	1.24 (−8.19, 10.73)	**MIN**	2.44 (0.04, 183.09)	1.16 (0.03, 44.26)	1.21 (0.02, 65.37)	0.32 (0.01, 25.79)	
	−0.87 (−8.14, 6.15)	2.35 (−4.21, 8.71)	1.09 (−10.23, 12.07)	**EN**	0.47 (0.04, 5.16)	0.50 (0.02, 11.82)	0.13 (0.01, 5.10)	
	−3.34 (−8.88, 1.78)	−0.12 (−5.01, 4.47)	−1.34 (−11.8, 8.53)	−2.47 (−9.02, 3.83)	**EF**	1.05 (0.11, 9.49)	0.28 (0.01, 5.42)	
	1.01 (−8.67, 10.62)	4.23 (−4.64, 13.17)	3.00 (−10.13, 15.95)	1.89 (−8.95, 13.08)	4.36 (−5.52, 14.67)	**P**	0.26 (0.02, 4.01)	
	–	–	–	–	–	–	**C**	
**Malunion**	**RIN**	1.67 (0.77, 4.35)	5.10 (0.80, 45.60)	2.34 (0.76, 8.33)	0.58 (0.01, 29.08)	0.41 (0.01, 9.39)	**Non-union**	
	1.06 (0.33, 3.22)	**UIN**	3.03 (0.52, 21.76)	1.39 (0.52, 3.86)	0.35 (0.01, 17.81)	0.24 (0.01, 5.87)		
	0.47 (0.05, 3.06)	0.44 (0.06, 2.51)	**EN**	0.45 (0.07, 2.69)	0.11 (0.01, 8.33)	0.08 (0.01, 3.10)		
	0.54 (0.16, 1.55)	0.51 (0.16, 1.46)	1.16 (0.20, 7.85)	**EF**	0.25 (0.01, 14.01)	0.17 (0.01, 4.81)		
	0.44 (0.05, 3.39)	0.41 (0.04, 3.74)	0.91 (0.06, 16.28)	0.79 (0.09, 7.10)	**P**	0.70 (0.07, 7.10)		
	2.29 (0.09, 131.63)	2.18 (0.07, 151.41)	5.05 (0.12, 492.75)	4.26 (0.14, 292.95)	5.53 (0.12, 518.01)	**C**		
**Implant failure**	**RIN**	0.73 (0.25, 2.16)	–	1.15 (0.10, 14.01)	2.80 (0.78, 9.39)	2.66 (0.27, 24.78)	0.12 (0.01, 6.89)	**Infection**
	0.32 (0.13, 0.62)	**UIN**	–	1.57 (0.16, 16.44)	**3.82 (1.20, 11.25)**	3.63 (0.34, 36.23)	0.17 (0.01, 9.68)	
	0.13 (0.01, 1.93)	0.42 (0.01, 7.24)	**MIN**	–	–	–	–	
	1.26 (0.06, 55.70)	3.97 (0.23, 177.68)	10.49 (0.16, 1808.04)	**EN**	2.44 (0.23, 22.87)	2.32 (0.09, 51.42)	0.11 (0.01, 10.18)	
	0.73 (0.20, 3.82)	2.32 (0.74, 12.18)	5.58 (0.30, 295.89)	0.59 (0.01, 17.46)	**EF**	0.95 (0.10, 8.94)	0.04 (0.01, 2.46)	
	0.36 (0.02, 8.25)	1.15 (0.07, 27.39)	2.83 (0.05, 330.30)	0.27 (0.01, 21.98)	0.49 (0.03, 6.62)	**P**	0.05 (0.01, 1.32)	
	–	–	–	–	–	–	**C**	

* *time to union is the mean difference value; other endpoints are odds ratio value*. *RIN, reamed intramedullary nailing; UIN, un-reamed intramedullary nailing; MIN, minimally reamed intramedullary nailing; EN, Ender nailing; EF, external fixation; P, plate; C, cast*. *Treatment plan and outcome indicators are bolded. The Bold parts indicate significant results*.

**Table 3:** Node-splitting results of the network meta-analysis for six efficacy endpoints.

**Comparison**	**Mean difference/odds ratio (95% CrI)**			***P*-value**
	**Direct**	**Indirect**	**Network**	**(Direct vs. indirect)**
TIME TO UNION^*^				
UIN vs. RIN	3.70 (−0.49, 8.40)	−0.01 (−12.00, 11.00)	3.20 (−0.61, 7.20)	0.474
EF vs. RIN	0.99 (−7.80, 9.30)	5.00 (−1.80, 12.00)	3.30 (−1.80, 8.80)	0.408
EN vs. UIN	−1.80 (−11.00, 7.30)	−3.00 (−15.00, 8.30)	−2.40 (−8.70, 3.90)	0.870
EF vs. UIN	1.30 (−5.20, 8.20)	−1.20 (−9.50, 6.40)	0.06 (−4.60, 4.70)	0.590
EF vs. EN	2.50 (−7.10, 11.00)	2.00 (−9.00, 13.00)	2.50 (−4.00, 8.80)	0.281
**REOPERATION**				
UIN vs. RIN	3.40 (1.20, 9.70)	0.30 (0.04, 2.40)	2.20 (0.82, 6.10)	**0.038**
EF vs. RIN	0.62 (0.10, 3.70)	4.50 (0.97, 23.00)	1.90 (0.54, 7.30)	0.098
P vs. RIN	1.30 (0.05, 56.00)	2.60 (0.11, 52.00)	1.90 (0.20, 16.00)	0.729
C vs. RIN	0.26 (0.01, 14.00)	1.00 (0.01, 83.00)	0.52 (0.03, 8.90)	0.641
EN vs. UIN	13.00 (0.44, 430.00)	0.26 (0.01, 7.50)	1.80 (0.17, 25.00)	0.085
EF vs. UIN	1.50 (0.33, 7.10)	0.41 (0.06, 2.80)	0.87 (0.29, 3.10)	0.280
EF vs. EN	2.60 (0.11, 54.00)	0.05 (0.01, 1.50)	0.47 (0.04, 5.30)	0.085
P vs. EF	2.50 (0.07, 56.00)	0.55 (0.02, 10.00)	1.00 (0.11, 8.90)	0.485
C vs. P	0.43 (0.01, 15.00)	0.10 (0.01, 11.00)	0.26 (0.02, 4.10)	0.575
**NON-UNION**				
UIN vs. RIN	1.80 (0.73, 5.50)	1.00 (0.10, 9.90)	1.70 (0.80, 4.10)	0.614
EF vs. RIN	1.60 (0.22, 13.00)	3.00 (0.74, 16.00)	2.30 (0.77, 8.00)	0.609
EN vs. UIN	9.10 (0.75, 350.00)	1.20 (0.08, 17.00)	3.30 (0.50, 22.00)	0.246
EF vs. UIN	1.30 (0.42, 5.00)	1.70 (0.25, 12.00)	1.40 (0.57, 3.80)	0.841
EF vs. EN	1.10 (0.10, 14.00)	0.13 (0.01, 2.60)	0.43 (0.06, 2.70)	0.285
**MALUNION**				
UIN vs. RIN	1.60 (0.47, 6.60)	0.34 (0.06, 2.10)	0.95 (0.32, 3.00)	0.148
EF vs. RIN	0.81 (0.27, 3.00)	6.90 (1.60, 36.00)	1.80 (0.73, 6.90)	**0.033**
P vs. RIN	8.50 (0.66, 490.00)	0.43 (0.01, 10.00)	2.30 (0.32, 19.00)	0.171
EN vs. UIN	11.00 (0.94, 240.00)	0.65 (0.07, 7.30)	2.20 (0.39, 18.00)	0.106
EF vs. UIN	2.30 (0.57, 11.00)	1.60 (0.29, 9.90)	1.90 (0.67, 6.50)	0.778
EF vs. EN	2.10 (0.22, 20.00)	0.13 (0.01, 2.50)	0.87 (0.14, 5.50)	0.105
P vs. EF	0.30 (0.01, 6.40)	6.30 (0.28, 340.00)	1.20 (0.14, 10.00)	0.161
**INFECTION**				
UIN vs. RIN	0.64 (0.18, 2.40)	1.00 (0.12, 11.00)	0.75 (0.26, 2.10)	0.681
EF vs. RIN	4.70 (0.67, 36.00)	1.90 (0.29, 9.60)	2.80 (0.81, 9.80)	0.440
P vs. RIN	1.20 (0.04, 35.00)	5.60 (0.25, 140.00)	2.60 (0.27, 24.00)	0.501
EN vs. UIN	10.00 (0.40, 530.00)	0.29 (0.01, 7.20)	1.40 (0.15, 16.00)	0.121
EF vs. UIN	2.40 (0.56, 9.70)	8.80 (1.20, 60.00)	3.70 (1.30, 11.00)	0.260
EF vs. EN	11.00 (0.46, 330.00)	0.32 (0.01, 9.80)	2.60 (0.25, 26.00)	0.125
P vs. EF	1.80 (0.09, 32.00)	0.36 (0.01, 14.00)	0.94 (0.10, 8.40)	0.456
**IMPLANT FAILURE**				
UIN vs. RIN	3.40 (1.70, 9.20)	0.60 (0.01, 26.00)	3.20 (1.70, 7.30)	0.344
EF vs. RIN	0.34 (0.01, 8.10)	1.80 (0.25, 9.50)	1.40 (0.25, 4.50)	0.358
EF vs. UIN	0.55 (0.09, 2.10)	0.10 (0.01, 2.10)	0.45 (0.08, 1.30)	0.344

* *Time to union is the mean difference value; other endpoints are odds ratio value*. *RIN, reamed intramedullary nailing; UIN, un-reamed intramedullary nailing; MIN, minimally reamed intramedullary nailing; EN, Ender nailing; EF, external fixation; P, plate; C, cast*. *The P-values is bolded show significant inconsistency between direct and indirect evidence*.

**Table 4:** Surface under the cumulative ranking curve (SUCRA) results of six efficacy endpoints in all cases.

**Target**	**Time to union**	**Reoperation**	**Non-union**	**Malunion**	**Infection**	**Implant failure**
RIN	**0.601**	**0.619**	**0.583**	0.539	0.455	**0.635**
UIN	0.214	0.324	0.373	**0.566**	**0.571**	0.239
MIN	0.374	0.427	–	–	–	0.150
EN	0.489	0.207	0.094	0.256	0.410	**0.601**
EF	0.213	0.377	0.255	0.264	0.136	0.535
P	**0.609**	0.366	0.546	0.246	0.193	0.340
C	–	**0.680**	**0.650**	**0.628**	**0.735**	–

^*^Treatment: RIN, reamed intramedullary nailing; UIN, un-reamed intramedullary nailing; MIN, minimally reamed intramedullary nailing; EN, Ender nailing; EF, external fixation; P, plate; C, cast.

^**^The place where the SUCRA value is bolded indicates the top 2 indicators.

### Reoperation

A remarkable difference on incidence of reoperation was not found either, in all 95% CrI including 1. C seemed to be most optimal, with the least occurrence of reoperation, since the OR value of C compared to the remaining six treatments was <1 (C vs. RIN OR: 0.52 95% CrI 0.03 to 8.76; C vs. MIN OR: 0.32 95% CrI 0.01 to 25.79; C vs. EF OR: 0.28 95% CrI 0.01 to 5.42; C vs. P OR: 0.26 95% CrI 0.02 to 4.01; C vs. UIN OR: 0.24 95% CrI 0.01 to 4.81; C vs. EN OR: 0.13 95% CrI 0.01 to 5.10), as shown in Table 2. Additionally, C (0.680) obtained the highest SUCRA value, followed by RIN (0.619) and MIN (0.427), as listed in Table 4. A significant inconsistency between direct and indirect data was discovered in UIN and RIN with *P*-value 0.038 (Table 3). Illustrated by the heat plot in Figure 3, this inconsistency derived the comparison between RIN, UIN, EN and EF, with an apparent warm color. No publication bias was present in the funnel plot in shown in Figure S2.

### Non-union

Non-union is a serious complication of a tibia shaft fracture, and it is most common after surgical treatment. Therefore, for C (C vs. P OR: 0.70 95% CrI 0.07 to 7.10; C vs. RIN OR: 0.41 95% CrI 0.01 to 9.39; C vs. UIN OR: 0.24 95% CrI 0.01 to 5.87; C vs. EF OR: 0.17 95% CrI 0.01 to 4.81; C vs. EN OR: 0.08 95% CrI 0.01 to 3.10) and P (P vs. RIN OR: 0.58 95% CrI 0.01 to 29.08; P vs. UIN OR: 0.35 95% CrI 0.01 to 17.81; P vs. EF OR: 0.11 95% CrI 0.01 to 8.33; P vs. EN OR: 0.25 95% CrI 0.01 to 14.01), the incidence of non-union was less than other interventions, even though its superiority was not significant, seen in Table 2. C (0.650) was still ranked at the top under non-union endpoint, but unlike the combined result, RIN (0.619) possessed a higher SUCRA value than P (0.546). Moreover, no remarkable inconsistency and publication bias was indicated (Figure 3 and Figure S2).

### Malunion

Malunion is another frequently occurring complication. Even though no intervention manifested an outstanding benefit, C (UIN vs. C OR: 2.18, 95% CrI 0.07 to 151.41; RIN vs. C OR: 2.29, 95% CrI 0.09 to 131.63; EF vs. C OR: 4.26, 95% CrI 0.14 to 292.95; EN vs. C OR: 5.05, 95% CrI 0.12 to 492.75; P vs. C OR: 5.53, 95% CrI 0.12 to 518.01) was relatively better than others. This was also verified by the SUCRA results in Table 4. The top three treatments in malunion were C (0.628), UIN (0.566) and RIN (0.539). A significant inconsistency was revealed by the Node-splitting table in Table 3, in the comparison between EN and RIN with *P*-value 0.033. Based on the information from the heat plot in Figure 3, the inconsistency of indirect data was mainly caused by RIN, UIN, EN, and EF. As to the publication bias, no obvious bias was found in Figure S2.

### Infection

Infection is a necessary concern for any treatment, especially for surgical procedures. Therefore, C (C vs. UIN OR: 0.17 95% CrI 0.01 to 9.68; C vs. RIN OR: 0.12 95% CrI 0.01 to 6.89; C vs. EN OR: 0.11 95% CrI 0.01 to 10.18; C vs. P OR: 0.05 95% CrI 0.01 to 1.32; C vs. EF OR: 0.04 95% CrI 0.01 to 2.46) was the treatment with the least reported cases of infection, including deep and superficial infection. While UIN (EF vs. UIN OR: 3.82 95% CrI 1.20 to 11.25) was the only other good treatment with statistical significance. As suggested by the SUCRA table in Table 4, C (0.735) was the optimal, followed by UIN (0.571) and RIN (0.455). A slight inconsistency was present among UIN, EN and EF in the heat plot in Figure 3. The publication related to the treatment of a tibia shaft fracture, on the outcome of an infection, is unbiased (Figure S2).

### Implant Failure

For the endpoint, the incidence of implant failure, EN (RIN vs. EN OR: 1.26 95% CrI 0.06 to 55.70; UIN vs. EN OR: 3.97 95% CrI 0.23 to 177.68; MIN vs. EN OR: 10.49 95% CrI 0.16 to 1,808.04; EN vs. EF OR: 0.59 95% CrI 0.01 to 17.46; EN vs. P OR: 0.27 95% CrI 0.01 to 21.98) outperformed all others, nevertheless, no significant difference was revealed. Patients treated with RIN (RIN vs. UIN OR: 0.32 95% CrI 0.13 to 0.62) had significantly fewer implant failures compared to UIN, as detailed in Table 2. The SUCRA rankings (Table 4) were as follows, RIN (0.635), EN (0.601), and EF (0.535). No distinct inconsistency existed according to the node-splitting table in Table 3 and the heat plot in Figure 3. Neither was the publication bias in Figure S2.

### Subgroup Analysis

Two subgroup analyses were conducted by including only open or closed tibia shaft fracture patients, the result of which are shown in Tables S2–S5.

## Discussion

In this Bayesian NMA, seven frequently used surgical and non-surgical treatments for open or closed shaft fractures of the tibia were assessed, in terms of time to union, the occurrence of reoperation, non-union, malunion, infection, and implant failure, with data provided by 25 RCTs and QRTs. The treatment methods of tibia fracture healing were reviewed and integrated. A total of seven methods were reviewed, without significant technical improvements. Unlike the former relevant meta-analysis which concentrated on the comparison of two specific interventions. Although a NMA in this field had been published, it mainly evaluated six surgical treatment options for open fractures of the tibia diaphysis, according to their performance on unplanned reoperation, malunion, deep infection and superficial infection which included 14 trials (Foote et al., 2015). Foote et al. produced a conclusion that intramedullary nailing might be more beneficial than other fixation strategies, in agreement with the viewpoint of our analysis. However, Foote et al. thought that UIN had the highest probability as the best treatment option for open tibia fractures, in conflict with our results that RIN might be a more suitable and advantageous treatment, regardless of C. We found that RIN ranked at the top three under four endpoints (time to union, reoperation, non-union, implant failure), while UIN performed better in malunion and infection.

RIN was also found to be very effective in both open and closed cases in the subgroup analysis. UIN had no significant effect in either closed or open cases. C remained the best treatment for closed cases in the case of three indices with supportive data. Considering the high risk of bias of trials in included Foote's NMA, and the outcome of some traditional meta-analyses that there was no significant difference between RIN and UIN for open cases, and RIN was recommended by more for closed fractures, we contend our conclusion that the type of fracture was not distinguished (Xue et al., 2010; Li et al., 2013; Shao et al., 2014; Xia et al., 2014; Yu et al., 2014).

Cast was the only non-surgical treatment method among the seven interventions. Two RCTs provided data, and most patients that received this treatment option were closed cases. The closed fracture was usually caused by a low energy accident, without the wound being exposed to air directly, which reduced the chance of infection and other surgery related complications. Therefore, it obtained a higher SUCRA score than other surgical treatments under infection endpoints (Van Der Linden and Larsson, 1979; Obremskey et al., 2017). The good performance of C under infection was also verified by our results, and as shown in Table 4, C (0.735) ranked top in infection. C was the ranked the best treatment in closed cases, as shown in Table 8. Related data was lacking in open cases, however, more RCT articles are required in order to evaluate its specific therapeutic effects.

RIN enjoyed the second highest probability as an effective alternative to C, and it was suggested in both open and closed tibia fractures, of numerous RCTs and meta-analyses. Intramedullary nailing functions via stabilization, depending on the contact between the stiff long bone and the elastic implant rod, and the area of this contact can be enlarged by reaming the medullary cavity. However, some serious adverse events may occur, such as bone necrosis, which could be a great restrictive factor (Pfister, 2010).

Unfortunately, this NMA also had some undeniable limitations. For several important treatments, more evidence was required to support their goodness of fit. In this analysis, C enjoyed the highest probability as the optimal treatment choice in closed cases. Nonetheless, only two firsthand comparisons meeting the criteria were included, as well as related data of “Time to union,” “Infection,” and “Implant failure.” For RIN, 325 more closed cases were researched than open cases. These uneven case distributions may influence the accuracy of the results. A balance of more high-quality RCTs concerning open fractures is therefore required.

Overall, cast might be the optimal treatment to reduce the incidence of reoperation, non-union, malunion and infection in closed cases, while reamed intramedullary nailing could be a good alternative option in both open and closed cases. However, this conclusion requires more RCTs to verify these results. If necessary, a subgroup analysis is also required to narrow the scope of treatment which may provide more specific suggestions. In clinical practice, patient's individual situation should be considered first and foremost.

## Author Contributions

HL, DY, and SW contributed to the conception and design of the study. DY, SW, and YZ analyzed and interpreted the data. HL drafted the article. LM critically revised the article for important intellectual content. All authors approved the final version of the article for publication.

### Conflict of Interest Statement

The authors declare that the research was conducted in the absence of any commercial or financial relationships that could be construed as a potential conflict of interest.

## Supplementary Material

Figure S1 Risk of bias graph and risk of bias summary.

Figure S2 Comparison adjusted funnel plots for publication bias analysis.

Table S1 Reasons for study exclusion.

Table S2 Network meta-analysis results for six efficacy endpoints in open cases.

Table S3 Network meta-analysis results for six efficacy endpoints in close cases.

Table S4 Surface under the cumulative ranking curve (SUCRA) results of six efficacy endpoints in open cases.

Table S5 Surface under the cumulative ranking curve (SUCRA) results of six efficacy endpoints in close cases.

## References

[B1] Alho A. Benterud J. G. Hogevold H. E. Ekeland A. Stromsoe K. (1992). Comparison of functional bracing and locked intramedullary nailing in the treatment of displaced tibial shaft fractures. Clin. Orthop. Relat. Res. 277, 243–250. 10.1097/00003086-199204000-00030 1555348

[B2] Alho A. Ekeland A. Stromsoe K. Benterud J. G. (1993). Nonunion of tibial shaft fractures treated with locked intramedullary nailing without bone grafting. J. Trauma 34, 62–67. 10.1097/00005373-199301000-00011 8437197

[B2a] Bach A. W. Hansen S. T. Jr. (1989). Plates versus external fixation in severe open tibial shaft fractures. A randomized trial. Clin. Orthop. Relat. Res. 241. 89–94. 2924483

[B3] Bhandari M. Guyatt G. Tornetta P. III Schemitsch E. H. Swiontkowski M. Sanders D. (2008). Randomized trial of reamed and unreamed intramedullary nailing of tibial shaft fractures: by the Study to Prospectively Evaluate Reamed Intramedullary Nails in Patients with Tibial Fractures (SPRINT) Investigators^*^. J. Bone Joint Surg. Am. 90, 2567–2578. 10.2106/JBJS.G.01694 19047701 PMC2663330

[B4] Blachut P. A. O'Brien P. J. Meek R. N. Broekhuyse H. M. (1997). Interlocking intramedullary nailing with and without reaming for the treatment of closed fractures of the tibial shaft. A prospective, randomized study. J. Bone Joint Surg. Am. 79, 640–646. 9160935 10.2106/00004623-199705000-00002

[B5] Braten M. Helland P. Grontvedt T. Aamodt A. Benum P. Molster A. (2005). External fixation versus locked intramedullary nailing in tibial shaft fractures: a prospective, randomised study of 78 patients. Arch. Orthop. Trauma Surg. 25, 21–26. 10.1007/s00402-004-0768-0 15611864

[B6] Busse J. W. Morton E. Lacchetti C. Guyatt G. H. Bhandari M. (2008). Current management of tibial shaft fractures: a survey of 450 Canadian orthopedic trauma surgeons. Acta Orthop. 79, 689–694. 10.1080/17453670810016722 18839377

[B7] Choudary D. Kanthimathi B. (2012). A prospective comparative study of reamed vs. unreamed nailing in fractures Shaft of Tibia. Malays Orthop. J. 6, 21–26. 10.5704/moj.1207.0016 25279051 PMC4093593

[B8] Court-Brown C. M. Mcbirnie J. (1995). The epidemiology of tibial fractures. J. Bone Joint Surg. Br. 77, 417–421. 10.1302/0301-620X.77B3.7744927 7744927

[B9] Court-Brown C. M. Will E. Christie J. McQueen M. M. (1996). Reamed or unreamed nailing for closed tibial fractures. A prospective study in Tscherne C1 fractures. J. Bone Joint Surg. Br. 78, 580–583. 8682824

[B10] Delee J. C. Stiehl J. B. (1981). Open tibia fracture with compartment syndrome. Clin. Orthop. Relat. Res. 160, 175–184. 10.1097/00003086-198110000-00027 7026116

[B11] Esan O. Ikem I. C. Orimolade E. A. Esan O. T. (2014). Implant failure in lower limb long bone diaphyseal fractures at a tertiary hospital in Ile- Ife. Nigeria. Niger. Postgrad. Med. J. 21, 181–184. 25126875

[B12] Fernandes H. J. Sakaki M. H. Silva Jdos S. dos Reis F. B. Zumiotti A. V. (2006). Comparative multicenter study of treatment of multi-fragmented tibial diaphyseal fractures with nonreamed interlocking nails and with bridging plates. Clinics 61, 333–338. 10.1590/S1807-59322006000400010 16924325

[B13] Finkemeier C. G. Schmidt A. H. Kyle R. F. Templeman D. C. Varecka T. F. (2000). A prospective, randomized study of intramedullary nails inserted with and without reaming for the treatment of open and closed fractures of the tibial shaft. J. Orthop. Trauma 14, 187–193. 10791670 10.1097/00005131-200003000-00007

[B14] Foote C. J. Guyatt G. H. Vignesh K. N. Mundi R. Chaudhry H. Heels-Ansdell D.. (2015). Which surgical treatment for open tibial shaft fractures results in the fewest reoperations? A Network Meta-analysis. Clin. Orthop. Relat. Res. 473, 2179–2192. 10.1007/s11999-015-4224-y 25724836 PMC4457757

[B15] Gaebler C. McQueen M. M. Vecsei V. Court-Brown C. M. (2011). Reamed versus minimally reamed nailing: a prospectively randomised study of 100 patients with closed fractures of the tibia. Injury 42, S17–S21. 10.1016/s0020-1383(11)70007-9 21939798

[B16] Grutter R. Cordey J. Buhler M. Johner R. Regazzoni P. (2000). The epidemiology of diaphyseal fractures of the tibia. Injury 31(Suppl. 3), C64–C67. 10.1016/S0020-1383(00)80035-2 11052384

[B17] Harris I. Lyons M. (2005). Reoperation rate in diaphyseal tibia fractures. ANZ J. Surg. 75, 1041–1044. 10.1111/j.1445-2197.2005.03618.x 16398806

[B18] Henley M. B. Chapman J. R. Agel J. Harvey E. J. Whorton A. M. Swiontkowski M. F. (1998). Treatment of type II, IIIA, and IIIB open fractures of the tibial shaft: a prospective comparison of unreamed interlocking intramedullary nails and half-pin external fixators. J. Orthop. Trauma 12, 1–7. 10.1097/00005131-199801000-00001 9447512

[B19] Holbrook J. L. Swiontkowski M. F. Sanders R. (1989). Treatment of open fractures of the tibial shaft: Ender nailing versus external fixation. A randomized, prospective comparison. J. Bone Joint Surg. Am. 71, 1231–1238. 2777852

[B20] Inan M. Halici M. Ayan I. Tuncel M. Karaoglu S. (2007). Treatment of type IIIA open fractures of tibial shaft with Ilizarov external fixator versus unreamed tibial nailing. Arch. Orthop. Trauma Surg. 127, 617–623. 10.1007/s00402-007-0332-9 17476515

[B21] Johner R. Wruhs O. (1983). Classification of tibial shaft fractures and correlation with results after rigid internal fixation. Clin. Orthop. Relat. Res. 178, 7–25. 10.1097/00003086-198309000-00003 6883870

[B22] Keating J. F. O'brien P. J. Blachut P. A. Meek R. N. Broekhuyse H. M. (1997). Locking intramedullary nailing with and without reaming for open fractures of the tibial shaft. A prospective, randomized study. J. Bone Joint Surg. Am. 79, 334–341. 10.2106/00004623-199703000-00003 9070520

[B23] Kulshrestha V. (2008). Incidence of infection after early intramedullary nailing of open tibial shaft fractures stabilized with pinless external fixators. Indian J. Orthop. 42, 401–409. 10.4103/0019-5413.43382 19753227 PMC2740348

[B24] Larsen L. B. Madsen J. E. Hoiness P. R. Ovre S. (2004). Should insertion of intramedullary nails for tibial fractures be with or without reaming? A prospective, randomized study with 3.8 years' follow-up. J. Orthop. Trauma 18, 144–149. 15091267 10.1097/00005131-200403000-00003

[B25] Li C. X. Zhao H. J. Zhao W. Q. Xu Y. Q. (2013). System evaluation on reamed and non-reamed intramedullary nailing in the treatment of closed tibial fracture. Acta Cir. Bras. 28, 744–750. 10.1590/S0102-86502013001000010 24114305

[B26] Littenberg B. Weinstein L. P. Mccarren M. Mead T. Swiontkowski M. F. Rudicel S. A.. (1998). Closed fractures of the tibial shaft. A meta-analysis of three methods of treatment. J. Bone Joint Surg. Am. 80, 174–183. 10.2106/00004623-199802000-00004 9486723

[B27] Milner S. A. Davis T. R. Muir K. R. Greenwood D. C. Doherty M. (2002). Long-term outcome after tibial shaft fracture: is malunion important? J. Bone Joint Surg. Am. 84-A, 971–980. 10.2106/00004623-200206000-00011 12063331

[B28] Mohseni M. A. Soleimanpour J. Mohammadpour H. Shahsavari A. (2011). AO tubular external fixation vs. unreamed intramedullary nailing in open grade IIIA-IIIB tibial shaft fractures: a single-center randomized clinical trial. Pak. J. Biol. Sci. 14, 490–495. 10.3923/pjbs.2011.490.495 21936253

[B29] Obremskey W. T. Cutrera N. Kidd C. M. Southeastern Fracture C. (2017). A prospective multi-center study of intramedullary nailing vs casting of stable tibial shaft fractures. J. Orthop. Traumatol. 18, 69–76. 10.1007/s10195-016-0429-4 27770336 PMC5311003

[B30] Pfister U. (2010). [Reamed intramedullary nailing]. Orthopade 39, 171–181. 10.1007/s00132-009-1522-7 20094703

[B31] Ramos T. Eriksson B. I. Karlsson J. Nistor L. (2014). Ilizarov external fixation or locked intramedullary nailing in diaphyseal tibial fractures: a randomized, prospective study of 58 consecutive patients. Arch. Orthop. Trauma Surg. 134, 793–802. 10.1007/s00402-014-1970-3 24664228

[B32] Rodrigues F. L. de Abreu L. C. Valenti V. E. Valente A. L. da Costa Pereira Cestari R. Pohl P. H. (2014). Bone tissue repair in patients with open diaphyseal tibial fracture treated with biplanar external fixation or reamed locked intramedullary nailing. Injury 45(Suppl. 5), S32–S35. 10.1016/s0020-1383(14)70018-x 25528622

[B33] Sadighi A. Elmi A. Jafari M. A. Sadeghifard V. Goldust M. (2011). Comparison study of therapeutic results of closed tibial shaft fracture with intramedullary nails inserted with and without reaming. Pak. J. Biol. Sci. 14, 950–953. 10.3923/pjbs.2011.950.953 22514897

[B34] Shao Y. Zou H. Chen S. Shan J. (2014). Meta-analysis of reamed versus unreamed intramedullary nailing for open tibial fractures. J. Orthop. Surg. Res. 9:74. 10.1186/s13018-014-0074-7 25149501 PMC4145248

[B35] Soleimanpour J. Feizi H. H. Mohseni M. A. Moradi A. Arzromchilar A. (2008). Comparison between ender and unreamed interlocking nails in tibial shaft fractures. Saudi Med J. 29, 1458–1462. 18946573

[B36] Swiontkowski M. F. Agel J. Mcandrew M. P. Burgess A. R. Mackenzie E. J. (2000). Outcome validation of the AO/OTA fracture classification system. J. Orthop. Trauma 14, 534–541. 10.1097/00005131-200011000-00003 11149498

[B37] Tabatabaei S. Arti H. Mahboobi A. (2012). Treatment of open tibial fractures: Comparison between unreamed and reamed nailing a prospective randomized trial. Pak. J. Med. Sci. 28:8.

[B37a] Tornetta P. Bergman M. Watnik N. Berkowitz G. Steuer J. (1994). Treatment of grade-IIIb open tibial fractures. A prospective randomized comparison of external fixation and non-reamed locked nailing. J. Bone Joint Surg. Br. 76, 13–19. 10.1302/0301-620X.76B1.8300656 8300656

[B38] Tu Y. K. Lin C. H. Su J. I. Hsu D. T. Chen R. J. (1995). Unreamed interlocking nail versus external fixator for open type III tibia fractures. J. Trauma 39, 361–367. 7674408 10.1097/00005373-199508000-00029

[B39] Vallier H. A. Cureton B. A. Patterson B. M. (2011). Randomized, prospective comparison of plate versus intramedullary nail fixation for distal tibia shaft fractures. J. Orthop Trauma 25, 736–741. 10.1097/BOT.0b013e318213f709 21904230

[B40] van der Linden W. Larsson K. (1979). Plate fixation versus conservative treatment of tibial shaft fractures. A randomized trial. J. Bone Joint Surg. Am. 61, 873–878. 383719

[B41] Van Der Linden W. Larsson K. (1979). Plate fixation versus conservative treatment of tibial shaft fractures. A randomized trial. J. Bone Joint. Surg. Am. 61, 873–878. 10.2106/00004623-197961060-00011 383719

[B42] Xia L. Zhou J. Zhang Y. Mei G. Jin D. (2014). A meta-analysis of reamed versus unreamed intramedullary nailing for the treatment of closed tibial fractures. Orthopedics 37, e332–e338. 10.3928/01477447-20140401-52 24762836

[B43] Xue D. Zheng Q. Li H. Qian S. Zhang B. Pan Z. (2010). Reamed and unreamed intramedullary nailing for the treatment of open and closed tibial fractures: a subgroup analysis of randomised trials. Int. Orthop. 34, 1307–1313. 10.1007/s00264-009-0895-x 19841919 PMC2989082

[B44] Yu G. S. Lin Y. B. Wang Y. Xu Z. Q. (2014). Reamed or unreamed intramedullary nailing for tibial fractures: a meta-analysis. Chin. J. Traumatol. 17, 229–234. 10.3760/cma.j.issn.1008-1275.2014.04.009 25098851

