# Supplementary figures and images for: Multiple Comparisons of the Efficacy and Safety for Seven Treatments in Tibia Shaft Fracture Patients
Source: Front Pharmacol. 2019 Apr 9;10:197. doi: 10.3389/fphar.2019.00197 (PMC6467001; doi:10.3389/fphar.2019.00197)

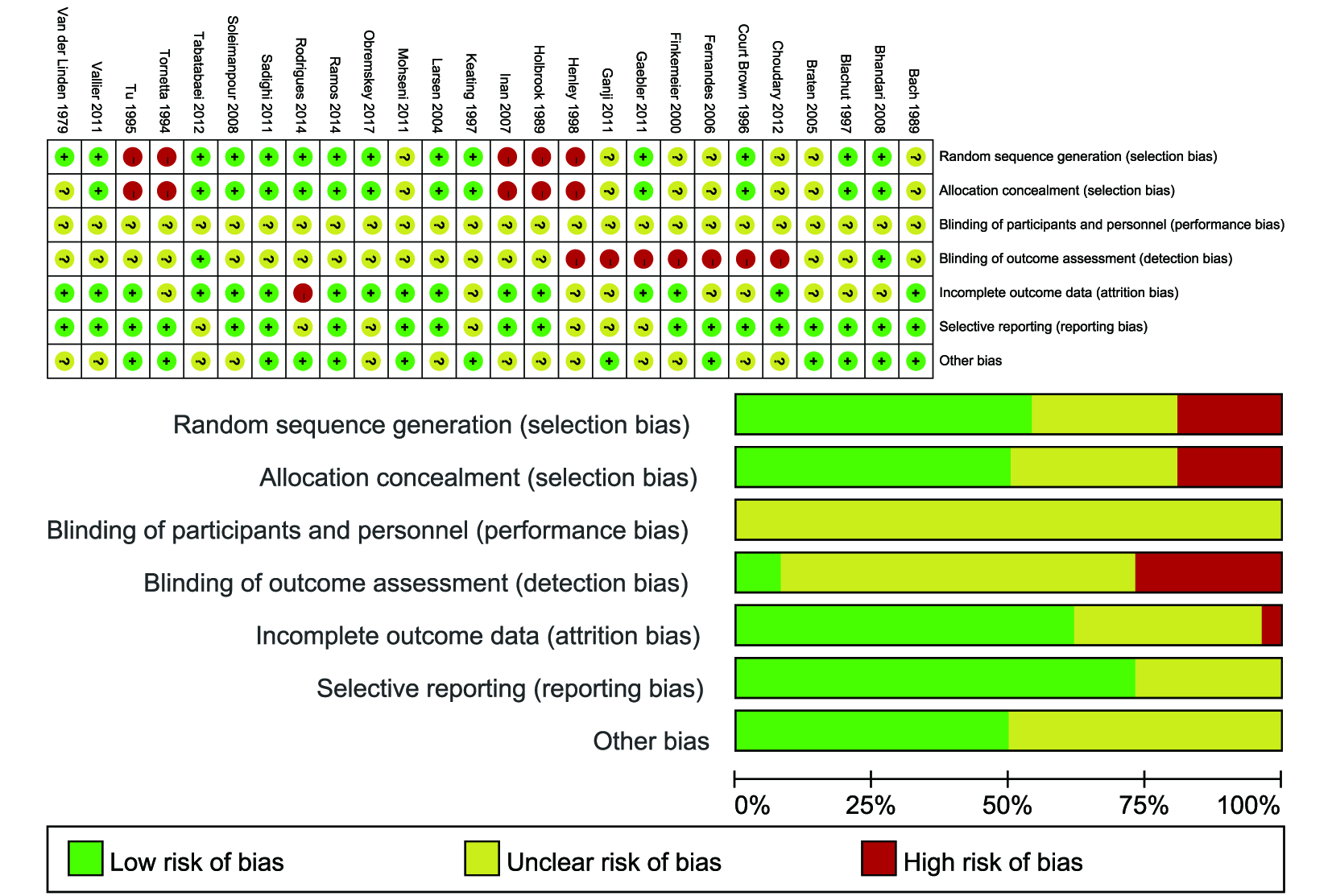

Supplement: Figure S1 — Risk of bias graph and risk of bias summary. [file Image_1.TIF]

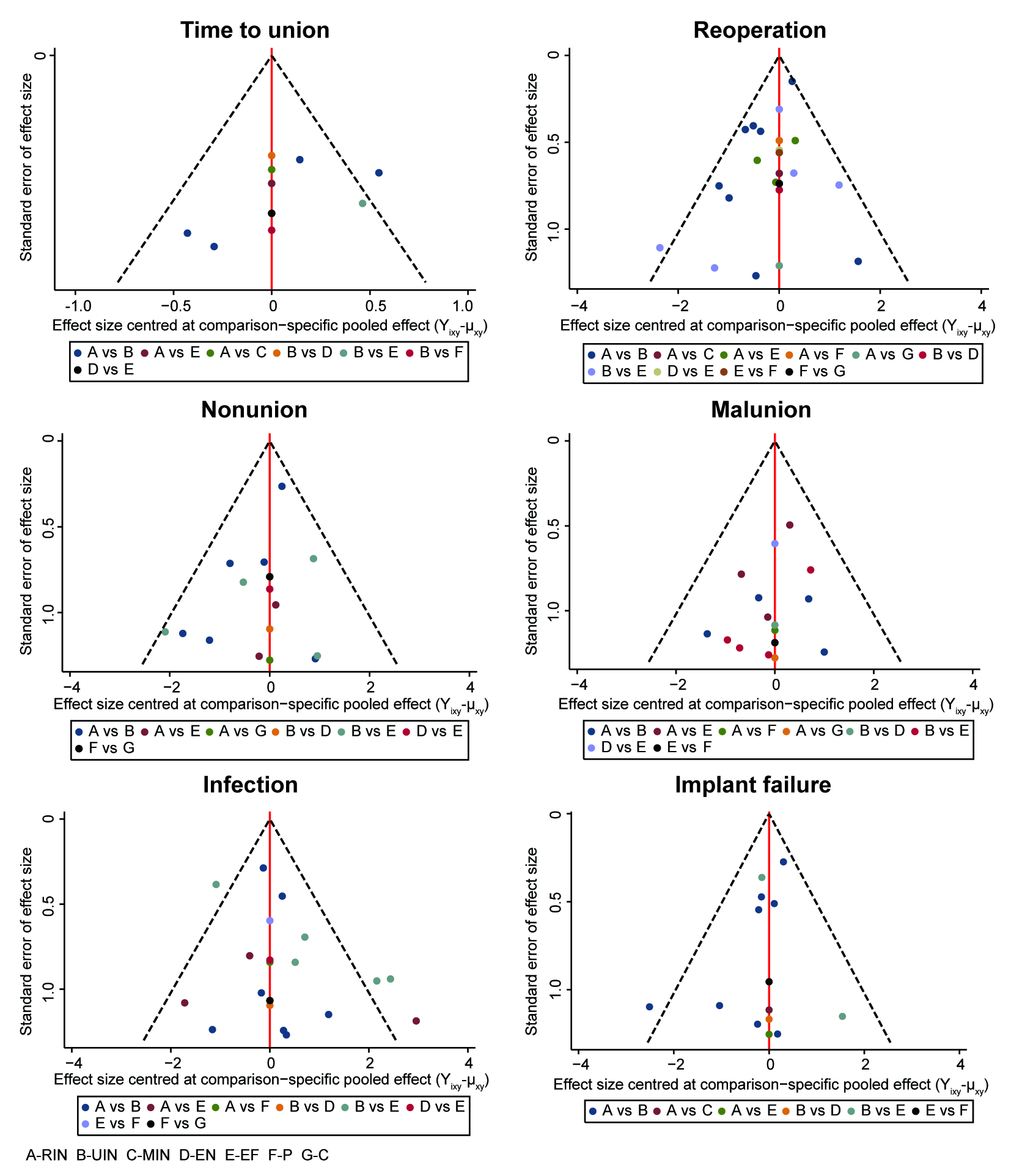

Supplement: Figure S2 — Comparison adjusted funnel plots for publication bias analysis. [file Image_2.TIF]
