# Supplementary material for: Multiple Comparisons of the Efficacy and Safety for Seven Treatments in Tibia Shaft Fracture Patients
Source: Front Pharmacol. 2019 Apr 9;10:197. doi: 10.3389/fphar.2019.00197 (PMC6467001; doi:10.3389/fphar.2019.00197)
Supplement: Table S1 — Reasons for study exclusion. [file Table_1.DOCX]

**Table S1. Reasons for study exclusion.**

| **Study** | | | |  |
| --- | --- | --- | --- | --- |
| **Author** | **Year** | **Country** | **Design** | **Reason for exclusion** |
| Toivanen et al. | 2002 | Finland | RCT | endpoints did not meet |
| Schemitsch et al. | 2012 | Canada | RCT | endpoints did not meet |
| Saied et al. | 2016 | India | Cohort comparative | design type did not meet |
| Pal et al. | 2015 | India | Comparative | design type did not meet |
| Mukherjee et al. | 2017 | India | Comparative | design type did not meet |
| O'Toole et al. | 2017 | USA | Prospective randomized trial | Publication type did not meet/No endpoints data provided |
| Nassif et al. | 2000 | USA | Prospective randomized trial | Publication type did not meet/No endpoints data provided |
| Antich-Adrover et al. | 1997 | Spain | Prospective randomized trial | endpoints did not meet |
| Bhandari et al. | 2008 | Canada | Prospective randomized trial | Publication type did not meet/No endpoints data provided |
| Birjandinejad et al. | 2009 | Iran | One-Arm | design type did not meet |
