# Supplementary material for: Multiple Comparisons of the Efficacy and Safety for Seven Treatments in Tibia Shaft Fracture Patients
Source: Front Pharmacol. 2019 Apr 9;10:197. doi: 10.3389/fphar.2019.00197 (PMC6467001; doi:10.3389/fphar.2019.00197)
Supplement: Table S2 — Network meta-analysis results for six efficacy endpoints in open cases. [file Table_2.DOCX]

**Table S2. Network meta-analysis results for six efficacy endpoints in open cases.**

| **Time to union*** | **RIN** | 0.65 (-0.81, 1.94) | -0.21 (-3, 2.6) | 0.74 (-0.75, 2.26) | 1.6 (-1.16, 4.39) | **Reoperation** |
| --- | --- | --- | --- | --- | --- | --- |
|  | -0.98 (-6.69, 5.04) | **UIN** | -0.85 (-3.34, 1.82) | 0.09 (-0.85, 1.24) | 0.96 (-1.51, 3.64) |  |
|  | 0.73 (-10.57, 11.65) | 1.73 (-8.08, 10.87) | **EN** | 0.95 (-1.38, 3.29) | 1.81 (-1.51, 5.15) |  |
|  | -2.07 (-10.33, 5.9) | -1.07 (-7.01, 4.25) | -2.78 (-10.41, 4.82) | **EF** | 0.86 (-1.48, 3.2) |  |
|  | - | - | - | - | **P** |  |
| **Nonunion** | **RIN** | -0.47 (-2.38, 1.32) | -0.5 (-2.94, 2.03) | 0.33 (-1.4, 2.07) | -0.94 (-5.09, 2.16) | **Malunion** |
|  | -0.25 (-2.31, 1.79) | **UIN** | -0.01 (-2.09, 2.19) | 0.8 (-0.31, 2.05) | -0.47 (-4.35, 2.44) |  |
|  | -0.46 (-4, 3.03) | -0.21 (-3.35, 2.91) | **EN** | 0.81 (-0.97, 2.61) | -0.48 (-4.63, 2.75) |  |
|  | -0.5 (-2.62, 1.56) | -0.25 (-1.6, 1.09) | -0.04 (-2.86, 2.78) | **EF** | -1.26 (-5.03, 1.36) |  |
|  | - | - | - | - | **P** |  |
| **Infection** | **RIN** | 1.11 (-0.22, 2.5) | - | 0.54 (-1.71, 2.32) | 1.18 (-2.21, 4.41) | **Implant failure** |
|  | 1.08 (-1.04, 3.29) | **UIN** | - | -0.57 (-2.32, 0.65) | 0.07 (-3.09, 3) |  |
|  | 2.75 (-1.39, 7.2) | 1.67 (-2.07, 5.68) | **EN** | 2.37 (-1.13, 6.05) | - |  |
|  | 0.36 (-1.86, 2.88) | -0.72 (-2.1, 0.84) | -2.37 (-6.05, 1.13) | **EF** | 0.65 (-1.91, 3.42) |  |
|  | -0.21 (-4.15, 4) | -1.29 (-4.8, 2.44) | -2.96 (-7.89, 1.87) | -0.58 (-3.87, 2.73) | **P** |  |

* Note: time to union is the mean difference value; other endpoints are odds ratio value.

** Treatment: RIN, reamed intramedullary nailing; UIN, un-reamed intramedullary nailing; MIN, minimally reamed intramedullary nailing; EN, Ender nailing; EF, external fixation; P, plate; C, cast.

*** Treatment plan and outcome indicators are bolded. The Bold parts indicate significant results
