# Supplementary material for: Multiple Comparisons of the Efficacy and Safety for Seven Treatments in Tibia Shaft Fracture Patients
Source: Front Pharmacol. 2019 Apr 9;10:197. doi: 10.3389/fphar.2019.00197 (PMC6467001; doi:10.3389/fphar.2019.00197)
Supplement: Table S3 — Network meta-analysis results for six efficacy endpoints in close cases. [file Table_3.DOCX]

**Table S3. Network meta-analysis results for six efficacy endpoints in close cases.**

| **Time to union*** | **RIN** | 1.84 (-2.5, 6.26) | 0.5 (-6.85, 7.92) | - | -1.49 (-9.35, 6.17) | **Reoperation** |
| --- | --- | --- | --- | --- | --- | --- |
|  | -7.37 (-17.07, 2.34) | **UIN** | -1.35 (-9.89, 7.26) | - | -3.32 (-12.34, 5.58) |  |
|  | -2.01 (-11.65, 7.73) | 5.38 (-8.27, 19) | **MIN** | - | -1.99 (-12.83, 8.7) |  |
|  | - | - | - | - | - |  |
|  | -3.06 (-16.74, 10.8) | 4.32 (-5.36, 14.28) | -1.04 (-17.66, 15.87) | **EF** | - |  |
|  | - | - | - | - | **C** |  |
| **Nonunion** | **RIN** | 1.54 (-0.21, 3.56) | - | - | -0.77 (-4.54, 2.47) | **Malunion** |
|  | -1.54 (-3.56, 0.21) | **UIN** | - | - | -2.35 (-6.56, 1.38) |  |
|  | - | - | **MIN** | - | - |  |
|  | - | - | - | **EF** | - |  |
|  | 0.77 (-2.47, 4.54) | 2.35 (-1.38, 6.56) | - | - | **C** |  |
| **Infection** | **RIN** | 1.74 (-0.55, 4.32) | 2.02 (-2.22, 6.79) | - | - | **Implant failure** |
|  | 0.5 (-1.34, 2.48) | **UIN** | 0.27 (-4.73, 5.52) | - | - |  |
|  | - | - | **MIN** | - | - |  |

* Note: time to union is the mean difference value; other endpoints are odds ratio value.

** Treatment: RIN, reamed intramedullary nailing; UIN, un-reamed intramedullary nailing; MIN, minimally reamed intramedullary nailing; EN, Ender nailing; EF, external fixation; P, plate; C, cast.

*** Treatment plan and outcome indicators are bolded. The Bold parts indicate significant results
