# Supplementary material for: Multiple Comparisons of the Efficacy and Safety for Seven Treatments in Tibia Shaft Fracture Patients
Source: Front Pharmacol. 2019 Apr 9;10:197. doi: 10.3389/fphar.2019.00197 (PMC6467001; doi:10.3389/fphar.2019.00197)
Supplement: Table S4 — Surface under the cumulative ranking curve (SUCRA) results of six efficacy endpoints in open cases. [file Table_4.DOCX]

**Table S4. Surface under the cumulative ranking curve (SUCRA) results of six efficacy endpoints in open cases.**

| **Target** | **Time to union** | **Reoperation** | **Nonunion** | **Malunion** | **Infection** | **Implant failure** |
| --- | --- | --- | --- | --- | --- | --- |
| **RIN** | 0.300 | 0.191 | 0.268 | **0.484** | **0.573** | 0.131 |
| **UIN** | **0.408** | 0.444 | 0.348 | 0.302 | 0.270 | **0.571** |
| **MIN** | - | - | - | - | - | **-** |
| **EN** | 0.245 | 0.184 | **0.415** | 0.325 | 0.087 | **-** |
| **EF** | **0.546** | **0.497** | **0.469** | **0.646** | 0.494 | 0.296 |
| **P** | - | **0.684** | - | 0.243 | **0.577** | 0.502 |
| **C** | - | - | - | - | **-** | - |

* Treatment: RIN, reamed intramedullary nailing; UIN, un-reamed intramedullary nailing; MIN, minimally reamed intramedullary nailing; EN, Ender nailing; EF, external fixation; P, plate; C, cast.

* * The place where the SUCRA value is bolded indicates the top 2 indicators.
