# Supplementary material for: Multiple Comparisons of the Efficacy and Safety for Seven Treatments in Tibia Shaft Fracture Patients
Source: Front Pharmacol. 2019 Apr 9;10:197. doi: 10.3389/fphar.2019.00197 (PMC6467001; doi:10.3389/fphar.2019.00197)
Supplement: Table S5 — Surface under the cumulative ranking curve (SUCRA) results of six efficacy endpoints in close cases. [file Table_5.DOCX]

**Table S5. Surface under the cumulative ranking curve (SUCRA) results of six efficacy endpoints in close cases.**

| **Target** | **Time to union** | **Reoperation** | **Nonunion** | **Malunion** | **Infection** | **Implant failure** |
| --- | --- | --- | --- | --- | --- | --- |
| **RIN** | **0.600** | **0.679** | 0.758 | 0.704 | 0.648 | **0.932** |
| **UIN** | 0.008 | 0.437 | 0.379 | 0.463 | **0.852** | 0.535 |
| **MIN** | 0.423 | 0.605 | - | - | - | 0.533 |
| **EN** | - | - | - | - | - | **-** |
| **EF** | - | - | - | - | - | - |
| **P** | **0.389** | - | - | - | - | - |
| **C** | - | **0.779** | **0.864** | **0.833** | **-** | - |

* Treatment: RIN, reamed intramedullary nailing; UIN, un-reamed intramedullary nailing; MIN, minimally reamed intramedullary nailing; EN, Ender nailing; EF, external fixation; P, plate; C, cast.

* * The place where the SUCRA value is bolded indicates the top 2 indicators.
